# Estrogen Promotes Endometrial Cancer Development by Modulating ZNF626, SLK, and RFWD3 Gene Expression and Inducing Immune Inflammatory Changes

**DOI:** 10.3390/biomedicines13020498

**Published:** 2025-02-17

**Authors:** Jiuming Fan, Mengyao Zhang, Huailiang Wu, Zehua Ye, Liyan Wang

**Affiliations:** 1 Department of Obstetrics and Gynecology, Renmin Hospital of Wuhan University, Wuhan 430060, China; fanjiuming16@henu.edu.cn; 2 Department of Critical Care Medicine, Renmin Hospital of Wuhan University, Wuhan 430060, China; zhangmydr@163.com; 3 Department of Anesthesiology, Renmin Hospital of Wuhan University, Wuhan 430060, China; whliang360@163.com

**Keywords:** endometrial cancer, estrogen, immune cell, bioinformatics analysis, gene expression omnibus, mendelian randomization

## Abstract

**Background**: Elevated estrogen has been found to contribute to the pathological development of endometrial cancer (EC), potentially through alterations in the tumor inflammatory immune microenvironment. However, the exact mechanisms underlying this process remain unclear. **Methods**: Bioinformatics was used to identify differentially expressed genes, analyze pathway enrichment, and assess their correlation with immune cell infiltration. Ishikawa cells and ECC-1 cells were stimulated with estradiol (E2) or the selective estrogen receptor modulator Arzoxifene, and qPCR was performed to measure gene expression changes. CCK8 and FACS assays were used to analyze cell cycle alterations, while Western blotting (WB) was used to evaluate apoptosis. **Results**: ZNF626 and SLK were highly expressed in EC tissues, whereas RFWD3 expression was downregulated. Immune cell infiltration analysis revealed a positive correlation between ZNF626 and M2 macrophages, while SLK was negatively correlated with M1 macrophages, memory B cells, and plasma cells. RFWD3 showed more complex correlations with multiple immune cell phenotypes, including T cells. E2 stimulation resulted in the increased expression of ZNF626 and SLK, while RFWD3 expression decreased. This was accompanied by enhanced cell proliferation and suppressed apoptosis. In contrast, Arzoxifene stimulation produced the opposite effects. **Conclusions**: Estrogen promotes cell proliferation and inhibits apoptosis by upregulating ZNF626 and SLK, while downregulating RFWD3. Furthermore, estrogen induces a shift in the tumor microenvironment, characterized by a reduction in memory CD4+ T cells and a transition from M1 to M2 macrophage phenotypes, thus facilitating the onset and progression of EC.

## 1. Introduction

Endometrial cancer (EC) is an epithelial malignant tumor that occurs in the endometrium and seriously affects the health of the female reproductive tract [1,2]. EC is strongly related to estrogen [3,4]. Estrogen regulates the hyperplasia, regeneration, and function of endometrial cells. It is related with the progression of EC either directly or through its influence on other hormones and metabolic pathways [5,6,7,8,9]. Prolonged exposure to excessive estrogen increases the mitosis of endometrial cells and the error rate of DNA replication [3]. The unopposed stimulation of the endometrium by estrogen is a well-established etiology associated with the development of this malignancy, a notion that has been widely corroborated [4,10,11]. Despite targeted therapies against estrogen demonstrating improved treatment outcomes in certain populations, tumors exhibiting specific morphological variants, adverse histopathological features, and/or advanced stages still display aggressive behavior and poor prognosis.

Consequently, further investigation into the mechanisms underlying endometrial cancer development is crucial for enhancing treatment prospects. Tumor occurrence results from the interplay between genetic and environmental factors. Much of the focus in the occurrence of EC has been on estrogen. However, the substantial effect of hormones on the tumor microenvironment and their interactions with genetic alterations involved in at least some mechanisms of endometrial cancer complicate its analysis [12,13,14,15,16,17]. The roles of estrogen downstream genes and their interactions with the inflammatory tumor microenvironment have not been fully elucidated. This study purposes to identify novel genes related to EC and carefully explore the potential connections between estrogen and these genes, while also investigating the interactions of these genes with the inflammatory tumor microenvironment to identify significant pathways for future research and potential targets for precision therapy in endometrial cancer.

## 2. Methods

**Figure 1:**
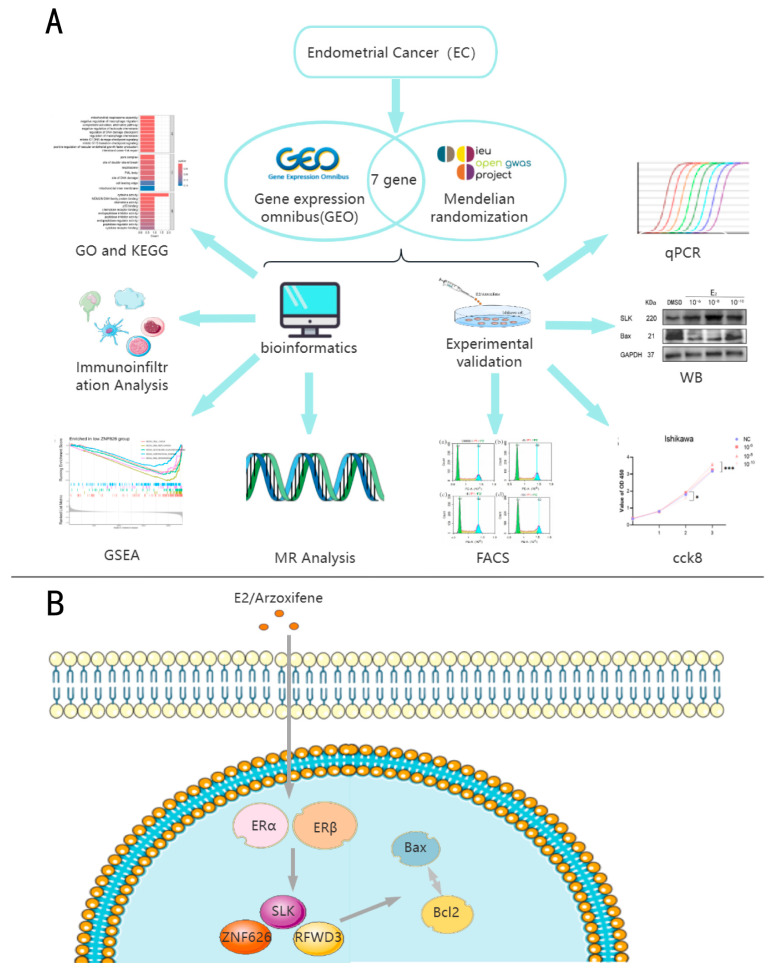
(**A**) Overview of this study. (**B**) The mechanisms by which E2 or Arzoxifene modulate cell proliferation and apoptosis were investigated. Bax and bcl2 are markers of apoptosis. * *p* < 0.05; *** *p* < 0.001.

The study design is shown in Figure 1.

### 2.1. Consent and Ethical Approval

These studies did not require ethical approval as they relied solely on public databases and commercially available, well-established cell lines.

### 2.2. Data Sources

The GEO database is a major resource for gene expression data [18]. We visited several EC-related microarray datasets, including GSE17025, GSE106191, and GSE120490. GSE106191 and GSE120490 were used as training sets, while GSE17025 was used for validation. The training sets were combined. The SVA package in R performed the correction for batch effects.

From GWAS, global genetic data linking variants to diseases and conditions, we selected data related to carcinoma in situ of the endometrium (excluding cancer cases), which included 201,459 samples with 137 cases. Additionally, we used 31,684 blood samples from eQTL Gen consortium (https://www.eqtlgen.org/; accessed on 13 October 2024) for two-sample mendelian randomization (MR) analysis.

### 2.3. Identification of Differential Genes

Gene names were assigned using Perl scripts and platform annotations. To adjust for background noise and normalize the data, we employed the limma package in R. Multiple anatomical site datasets were integrated using the sa package in R to correct for batch effects [19]. Datasets before and after removal of the batch effect were visualized using principal component analysis (PCA). The merged dataset was then used for subsequent analyses.

To analyze variant genes between normal and EC samples, the limma package was again applied in R. Bayesian methods were used to compute t-values, f-values, and log odds ratios. Genes with |logFC| ≥ 0.3 and an adjusted *p*-value of less than 0.05 were recognized as differentially expressed genes (DEGs). To visualize the results, we used ggplot2 and pheatmap to generate volcano and heat maps.

### 2.4. Mendelian Randomization

We used a two-sample MR approach to explore causal relationships between gene expression and EC, with single nucleotide polymorphisms (SNPs) as instrumental variables (IVs). Data of genes were sourced from GWAS, while data from eQTL were used as exposure information.

MR was performed using the “Two-Sample MR” package in R, applying inverse variance weighting (IVW) to evaluate the relationship between gene levels and EC risk. Genes with inconsistent odds ratios across multiple methods were removed. Sensitivity analyses for heterogeneity, pleiotropy, and Leave-One-Out (LOO) testing were also performed to ensure result robustness, particularly focusing on horizontal pleiotropy, excluding results with *p*-values < 0.05.

### 2.5. Bioinformatics Analysis

#### 2.5.1. GO and KEGG Analyses

We conducted Gene Ontology (GO) and Kyoto Encyclopedia of Genes and Genomes (KEGG) pathway analyses to explore the biological roles of differentially expressed genes (DEGs) using the ClusterProfiler R package (v3.20) [20]. GO analysis was applied to biological processes (BPs), cellular components (CCs), and molecular functions (MFs). Enrichment results were considered significant with a *p*-value < 0.05.

#### 2.5.2. Immune Cell Analysis

CIBERSORT was used to analyze the infiltration and relevance of 22 immune cell types in EC, providing insight into the immune landscape of the disease [21].

#### 2.5.3. GSEA

Gene Set Enrichment Analysis (GSEA) was used to assess the significance of gene expression by focusing on entire gene sets rather than individual genes. This method identifies key biological processes or pathways associated with EC, offering high sensitivity and reliability. We applied GSEA to examine two key DEGs and their roles in biological functions and pathways in the low-expression group of EC.

### 2.6. In Vitro Validation of Differentially Expressed Genes

#### 2.6.1. Cell Culture

The ECC-1 cell line was sourced from Xiamen Yimo Bio (Xiamen, China), while Ishikawa cells were obtained from Procell Life Science and Technology (Wuhan, China), and both cell lines were confirmed to be free of contamination. DMEM medium (SH30022.01, Hyclone, Logan, UT, USA) supplemented with 10% fetal bovine serum (FBS) (C04001-050X10, VivaCell, Shanghai, China) and 1% penicillin/streptomycin (BL505A, Biosharp, Hefei, China) under conditions of 37 °C and 5% CO_2_ was used to culture Ishikawa cells and ECC-1 cells.

#### 2.6.2. Drug Intervention

E2 (E8875, Sigma-Aldrich, St. Louis, MO, USA) and Arzoxifene (a selective estrogen receptor modulator) (LY353381, MedChemExpress, Monmouth Junction, NJ, USA) were dissolved in DMSO. Ishikawa cells and ECC-1 cells were treated with varying concentrations of E2 (0 [control], 10^−6^ mol/L, 10^−8^ mol/L, and 10^−10^ mol/L) and Arzoxifene (0 [control], 10^−3^ mol/L, 10^−4^ mol/L, and 10^−5^ mol/L) for incubation durations of 48 h and 72 h, separately.

#### 2.6.3. Cell Viability Detection

Transfected cells were plated in 96-well plates at a density of 2 × 10^3^ cells per well. A total of 10 μL of Cell Counting Kit-8 (CCK-8) reagent (CK04, Dojindo, Mashiki, Japan) was added to each well, followed by incubation of the cells at 37 °C and 5% CO_2_ for 1 h. Detection was conducted using a microplate spectrophotometer. Following seeding, detection was conducted on days 1, 2, and 3.

#### 2.6.4. Cell Cycle Analysis

According to the manufacturer’s instructions, for cell cycle distribution analysis, cells were collected and stained with propidium iodide from a Cell Cycle Staining Kit (LiankeBio, Hangzhou, China) at room temperature for 30 min. The cell cycle distribution was then analyzed using flow cytometry (NovoCyte 3000, Agilent Technologies, Santa Clara, CA, USA). The experiment was performed in triplicates and repeated three times.

#### 2.6.5. Real-Time Quantitative Polymerase Chain Reaction (RT-qPCR)

The culture of Ishikawa cells and ECC-1 cells was carried out under the conditions of a density of 2 × 10^5^ cells per well in 6-well plates and assigned to control and experimental groups. The control group was processed with DMSO and in a high-glycemic environment, whereas the experimental group received E2 for 48 h or Arzoxifene for 72 h. Total RNA was isolated using TRIzol reagent (TaKaRa, Dalian, China), according to the manufacturer’s instructions. RT-qPCR was conducted using SYBR Ex Taq Premix (TaKaRa). The results were normalized to the expression levels of GAPDH and quantified using the 2^−ΔΔCt^ method. The primer sequences are detailed in Table 1.

#### 2.6.6. Western Blot Analysis

Proteins were extracted from cells subjected to RAPI lysis after treatment with E2 for 48 h or Arzoxifene for 72 h. The antibodies employed in this study included rabbit anti-GAPDH (1:10,000, Proteintech, Wuhan, China), rabbit anti-SLK (1:1000, Proteintech, Wuhan, China), and rabbit anti-Bax (1:1000, Proteintech, Wuhan, China). GAPDH served as an internal control. Subsequently, Western blot analysis was conducted.

### 2.7. Statistical Analysis

Statistical analyses were conducted using SPSS 26.0, GraphPad Prism 9.0, and R software (v4.1.3). All in vitro experimental data are presented as the mean ± standard deviation (SD) derived from a minimum of three independent experiments. Group comparisons were conducted according to data type: comparisons between two groups utilized an unpaired two-tailed Student’s *t*-test, whereas comparisons among multiple groups employed one-way analysis of variance (ANOVA). Post hoc analyses following ANOVA were performed using Tukey’s multiple comparison test to mitigate the inflated false positive rate inherent in multiple testing. Statistical significance thresholds were defined as *p* < 0.05, *p* < 0.01, and *p* < 0.001, with “ns” denoting no statistical significance. Applying multiple comparison corrections ensured robust and reliable statistical inferences, particularly in multi-group analyses, thereby minimizing the risk of Type I errors.

## 3. Results

### 3.1. GEO Data Processing

**Figure 2:**
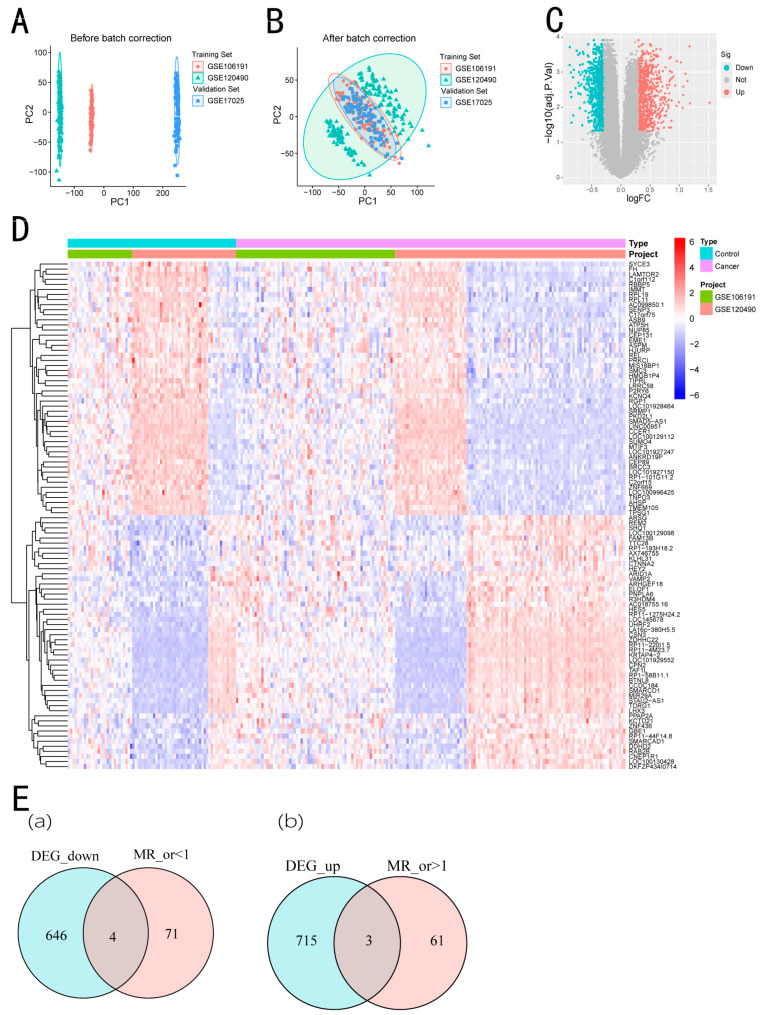
The gene differential expression analysis of GSE106191 and GSE120490 datasets. (**A**) The two-dimensional PCA cluster plot shows the differences before the batch effect is eliminated in the sample. (**B**) The two-dimensional PCA cluster plot shows the difference after the batch effect is eliminated in the sample. (**C**) The DEG volcano map shows upregulated genes in red and downregulated genes in green. (**D**) Heat map showing the expression of the top 100 differentially expressed genes (DEGs). (**E**) Venn diagram showing seven overlapping genes between differentially expressed genes (DEGs) and MR-identified causal genes, suggesting their potential involvement in EC. (**a**) Overlapped differential genes with increased gene expression; (**b**) Overlapped differential genes with decreased gene expression.

We integrated two datasets, GSE106191 and GSE120490, comprising 33 normal samples and 209 EC samples. We analyzed the gene expression levels of two samples before and after batch-effect correction, as well as PCA (Figure 2A,B).

### 3.2. Identification of Differentially Expressed Genes

Using the limma package in R, we identified 1368 differentially expressed genes (DEGs) related to EC from the intersection of the GSE106191 and GSE120490 datasets, with 650 genes upregulated and 718 genes downregulated in EC. A list of DEGs related to EC could be seen Supplementary Table S1 for details. Genetic differences were visualized in heat maps and volcano maps (Figure 2C,D).

### 3.3. Genome-Wide MR Analysis

A total of 31,684 samples were selected from eQTL Gen consortium as exposure factors and EC as an outcome factor to conduct two-sample MR analysis (Supplementary Table S2). This analysis aimed to identify genes with a potential causal relationship with EC, utilizing genetic variants as instrumental variables to reduce confounding effects and reverse causality. The results were primarily based on the IVW method, with a significance threshold set at *p* < 0.05. The IVW method provides a weighted average of causal estimates from multiple genetic instruments, ensuring more robust and reliable conclusions. Additionally, by removing genes with inconsistent odds ratios across five statistical methods and those showing horizontal pleiotropy (*p* < 0.05), 139 genes with a causal link with EC were identified (Supplementary Table S3).

### 3.4. Acquisition of Intersecting Genes

We cross-analyzed the 1368 DEGs with 139 genes from MR analysis. This step was performed to identify key genes that are not only differentially expressed in EC but also exhibit a potential causal association, providing stronger evidence for their involvement in EC pathogenesis. A Venn diagram was used to visualize the intersection, revealing seven genes present in both groups: ZNF544, ZNF626, SLK, HIGD2A, RFWD3, C5, and SECTM1 (Figure 2E, Supplementary Tables S4 and S5).

### 3.5. Function Enrichment Analysis of Intersecting Genes

**Figure 3:**
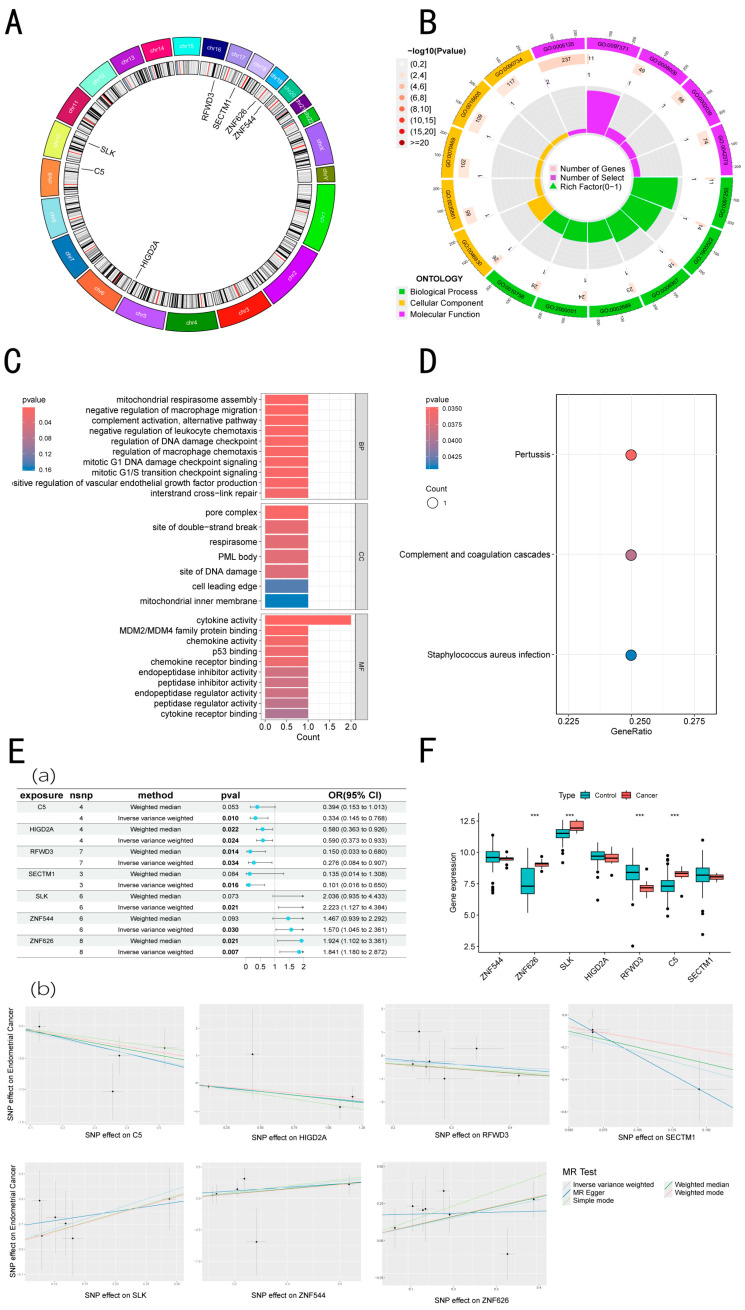
Functional enrichment analysis of five intersecting genes and MR analysis of seven intersecting genes were performed to explore their roles in these processes. (**A**) The chromosomal loop of seven intersecting genes. (**B**) GO circle map of seven intersecting genes. (**C**) The bubble map of the GO of seven intersecting genes. (**D**) The KEGG bubble map of seven intersecting genes. (**E**) MR results of 7 genes and EC. (**a**) MR forest map of seven core genes and EC. (**b**) The causal effect diagrams of 7 genes and EC are displayed by 5 methods. (**F**) Seven intersecting genes validated in the GSE17025 dataset. *** *p* < 0.001.

The seven genes obtained were analyzed by bioinformatics methods. A circular plot of chromosomes illustrated the locations of ZNF544, ZNF626, SLK, HIGD2A, RFWD3, C5, and SECTM1, located separately on chromosomes 19, 19, 10, 5, 16, 9, and 17 (Figure 3B). Our GO term analysis clearly indicates that ZNF544, ZNF626, SLK, HIGD2A, RFWD3, C5, and SECTM1 participate in diverse physiological processes, including mitochondrial respirasome assembly, the negative regulation of macrophage migration, complement activation pathways, negative modulation of leukocyte migration, regulation of the DNA damage checkpoint, regulation of macrophage chemotaxis, and mitotic G1 DNA damage checkpoint signaling. Cellular components include the pore complex, sites of double-strand break, respirasome, PML body, and sites of DNA damage. Functions related to the physiological activities of genes include cytokine activity, MDM2/MDM4 family protein binding, chemokine activity, p53 binding, and chemokine receptor binding (Figure 3A,C, Supplementary Table S6). An enrichment analysis of appellate genes clarified their key functions, including the modulation of metabolism, hyperplasia, and emergency response. Furthermore, the figure also emphasizes the role of complement and coagulation cascades in cancer (Figure 3D).

### 3.6. Single-Gene MR Analysis

In two-sample MR analysis, the above seven genes were used as independent variables, and EC was used as the dependent variable. Our findings indicate that the genes ZNF544, ZNF626, and SLK promote EC disease, whereas HIGD2A, RFWD3, C5, and SECTM1 exhibit protective effects (Figure 3E). By analyzing the gene expression trends related to EC obtained from the GEO dataset, we found that the expression levels of HIGD2A, RFWD3, C5, and SECTM1 were lower in EC patients compared to normal controls, indicating a potential protective role in EC pathology.

### 3.7. Gene Expression in GSE17025

In GSE17025, we detected the expression levels of ZNF544, ZNF626, SLK, HIGD2A, RFWD3, C5, and SECTM1, where the statistically significant genes showing trends consistent with the training set included ZNF626, SLK, and RFWD3. Compared to healthy individuals, the expression levels of ZNF626 and SLK were elevated in EC patients, suggesting that these genes may promote tumor occurrence and development. Conversely, RFWD3 exhibited a decreased expression in tumor patients (Figure 3F).

### 3.8. Immunohistochemical Analysis of ZNF626, SLK, and RFWD3 Expression in Endometrial Cancer

**Figure 4:**
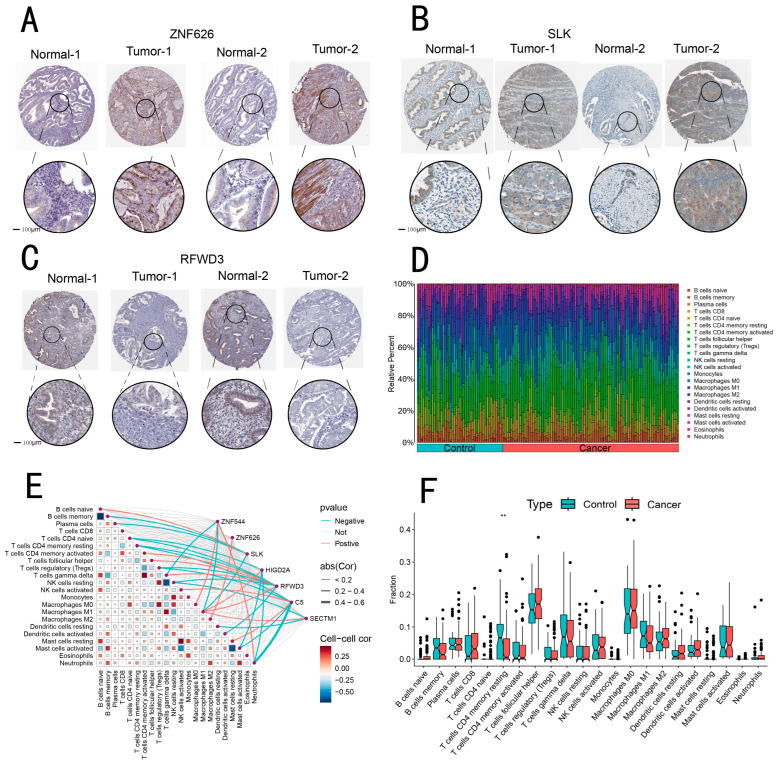
Immunohistochemistry and immune infiltration analyses. (**A**–**C**) The local expression patterns of ZNF626, SLK, and RFWD3 in tissue samples were further analyzed using the Human Protein Atlas (HPA) database. (**D**) The amount of 22 immune cells in the normal group and the experimental group. (**E**) The difference in immune cell expression between the normal group and experimental group. (**F**) The correlation analysis of seven core genes and immune cells. ** *p* < 0.01.

To validate the differential expression of ZNF626, SLK, and RFWD3 proteins in endometrial cancer (EC), we compared their expression levels in EC tissues with those in normal endometrial tissues. Immunohistochemical data from the HPA database revealed elevated levels of ZNF626 and SLK proteins in EC tissues, while RFWD3 expression was significantly reduced. Representative immunohistochemical images are shown in Figure 4A–C. These findings provide compelling evidence for the overexpression of ZNF626 and SLK, as well as the downregulation of RFWD3 in EC, suggesting their potential as therapeutic targets.

### 3.9. Immunocellular Analysis

We analyzed the relationship of relevant genes and 22 immune cells. We first compared the relative proportions of different types of immune cells. The changes in two datasets indicated that the composition of immune cell groups underwent significant alterations due to experimental intervention. For instance, the ratio of CD4+ memory resting T cells decreased in the tumor group, indicating that tumor cells may reduce the immune capacity of the body through immune evasion mechanisms, alterations in the immune microenvironment, and exhaustion due to chronic antigen stimulation (Figure 4D, Supplementary Table S7). Correlation analysis revealed a positive correlation between M1 macrophages and genes HIGD2A, RFWD3, and SECTM1, whereas a negative correlation in ZNF544 and SLK. ZNF626 and M2 macrophages were found to have a positive correlation only. RFWD3 was related to multiple immune cell populations. C5 showed a strong negative correlation with neutrophils (Figure 4F). In our sample data, CD4 memory resting T cells exhibited statistical significance (Figure 4E). HIGD2A, RFWD3, and SECTM1 may also alter the immune response of tumors in the body by affecting the hyperplasia and apoptosis of macrophages.

### 3.10. Single-Gene GSEA

**Figure 5:**
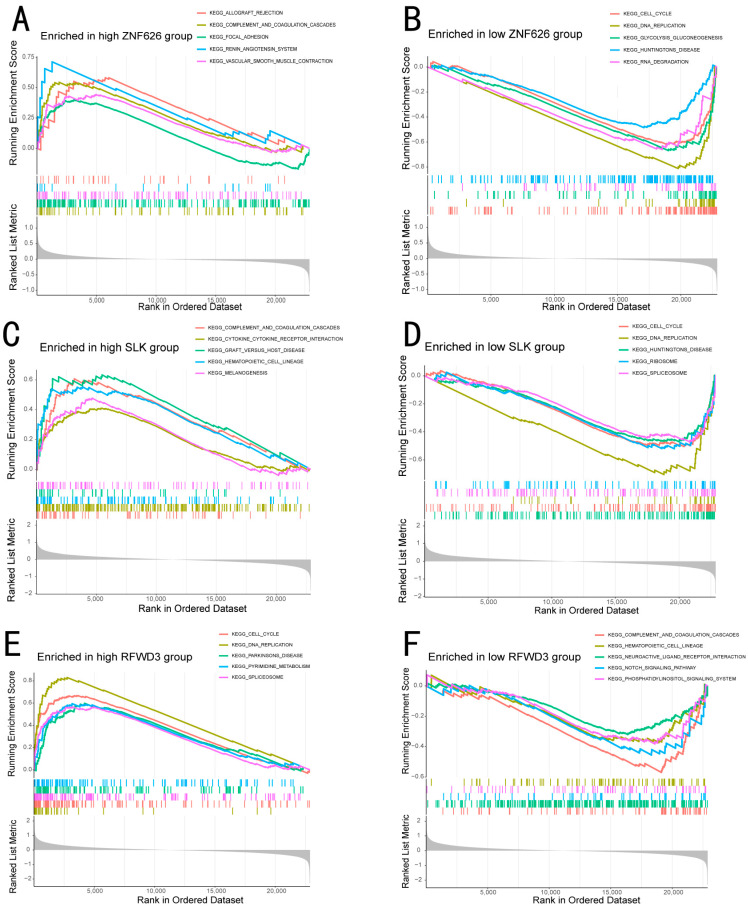
GSEA enrichment analysis of 3 DEGs. (**A**) The KEGG curve was enriched in the high-ZNF626 group. (**B**) The KEGG curve was enriched in the low-ZNF626 group. (**C**) The KEGG curve was enriched in the high-SLK group. (**D**) The KEGG curve was enriched in the low-SLK group. (**E**) The KEGG curve was enriched in the high-RFWD3 group. (**F**) The KEGG curve was enriched in the low-RFWD3 group.

GSEAs were used to study the biological function of the studied genes. We selected ZNF626, SLK, and RFWD3 (Supplementary Tables S8–S10), which showed statistical significance in the validation set and were aligned with trends in the training set. In the increased ZNF626 and SLK and decreased RFWD3 EC samples, the complement and coagulation cascades were notably active (Figure 5A,C,F). Tumor cells may escape by altering the immune function of the body through the complement system. In the decreased ZNF626 and increased SLK samples, the cell cycle and DNA replication pathways were significantly activated (Figure 5B,D,E). Similarly, these three genes are likely to promote the division and proliferation of tumor cells by altering certain molecules involved in DNA replication and the cell cycle.

### 3.11. E2 Promotes Cell Proliferation in the Ishikawa Cell Line and ECC-1 Cell Line

**Figure 6:**
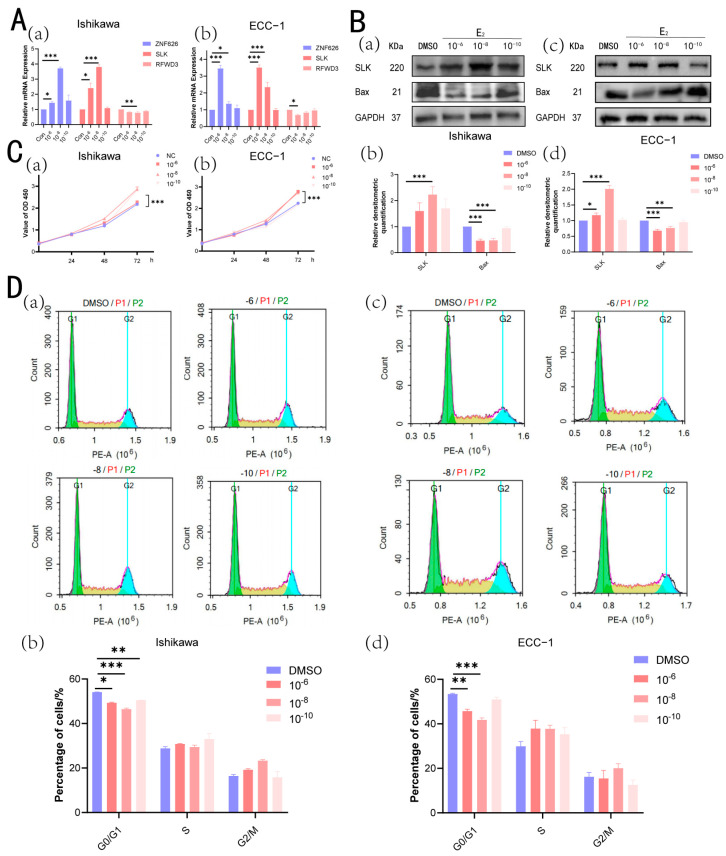
E2 enhances the proliferation and apoptosis of Ishikawa cells and ECC-1 cells. (**A**) The relative expression levels of ZNF626, SLK, and RFWD3 in Ishikawa cells (**a**) and ECC-1 cells (**b**) were assessed using qRT-PCR following stimulation with various concentrations of E2 (0 [control], 10^−6^ mol/L, 10^−8^ mol/L, and 10^−10^ mol/L) for 48 h. (**B**) Western blot analysis of SLK and Bax expression levels in Ishikawa cells (**a**) and ECC-1 cells (**c**) after 48 h of stimulation with varying E2 concentrations. Corresponding histograms present the quantified Western blot results for Ishikawa cells (**b**) and ECC-1 cells (**d**). (**C**) CCK8 assays demonstrated the effects of different E2 concentrations on the proliferation of Ishikawa cells (**a**) and ECC-1 cells (**b**) at 24, 48, and 72 h. (**D**) The percentage distribution of cells across different cell cycle phases in Ishikawa cells (**a**) and ECC-1 cells (**c**) after 48 h of stimulation with varying E2 concentrations. A histogram illustrating the average percentages of cells in the G0/G1, S, and G2/M phases for both control and treated groups in Ishikawa cells (**b**) and ECC-1 cells (**d**). Values represent the mean ± S.E.M. of a minimum of three independent experiments. Significance is calculated by performing an unpaired *t*-test between the control and treated groups: * *p* < 0.05; ** *p* < 0.01; *** *p* < 0.001.

The Ishikawa cell line and ECC-1 cell line, known for their high sensitivity to E2, were stimulated using varying concentrations of E2. Quantitative PCR (qPCR) was employed to evaluate the expression levels of ZNF626, SLK, and RFWD3 because these genes exhibited significant differential expression, with consistent trends observed in both the validation and training sets (Figure 6A). We found that stimulating Ishikawa cells with 10^−8^ mol/L E2 resulted in approximately a fourfold increase in the mRNA expression of ZNF626 and SLK, while RFWD3 expression decreased by about 20%. At the protein level, the validation of SLK showed that its expression increased by about twofold when Ishikawa cells were stimulated with 10^−8^ mol/L E2, consistent with our findings from bioinformatics and qPCR (Figure 6B). Additionally, we validated cell viability and cell cycle function by stimulating Ishikawa cells with different concentrations of E2 (Figure 6C,D). On the third day after stimulation with 10^−8^ mol/L E2, the CCK-8 assay showed statistically significant differences compared to the NC group, indicating notable cell proliferation. The results of cell cycle analysis also showed that compared with the NC group, the percentage of cells in the G0/G1 phase was significantly decreased after 48 h of treatment with 10 μmol/L of E2, indicating that E2 may promote the transition of cells from the G1 phase to S phase, accelerate the cell cycle process, and thus enhance cell proliferation. This phenomenon is consistent with the role of E2 in promoting cell growth, further supporting its potential role in the proliferation of endometrial cancer cells. Interestingly, in Figure 6A,C,D, the middle concentration (10^−8^ mol/L) exhibited the highest response. This may be due to a dose-dependent biphasic effect of E2. At moderate concentrations, E2 optimally activates estrogen receptors (ERs), leading to peak gene expression and cell proliferation. However, at higher concentrations (10^−6^ mol/L), receptor saturation or feedback inhibition may reduce the response, while at lower concentrations (10^−10^ mol/L), the stimulation might not be strong enough. We speculate that the cells might also be affected in terms of apoptosis, so we examined the expression of the Bax protein, which is known to promote apoptosis. The results showed that at 10^−8^ mol/L, the expression of Bax was reduced by approximately 50%, indicating that apoptosis was inhibited. ECC-1 cells exhibited similar effects; however, due to differences in estrogen sensitivity, the two cell lines showed distinct responses. The estrogen concentrations required to induce significant changes in gene expression and marked alterations in cell cycle and apoptosis differed between Ishikawa and ECC-1 cells. Overall, under appropriate concentrations of estrogen stimulation, estrogen can alter cell proliferation, cycle progression, and apoptosis by promoting the expression of significantly different genes.

### 3.12. Arzoxifene Inhibits Cell Proliferation in the Ishikawa Cell Line and ECC-1 Cell Line

**Figure 7:**
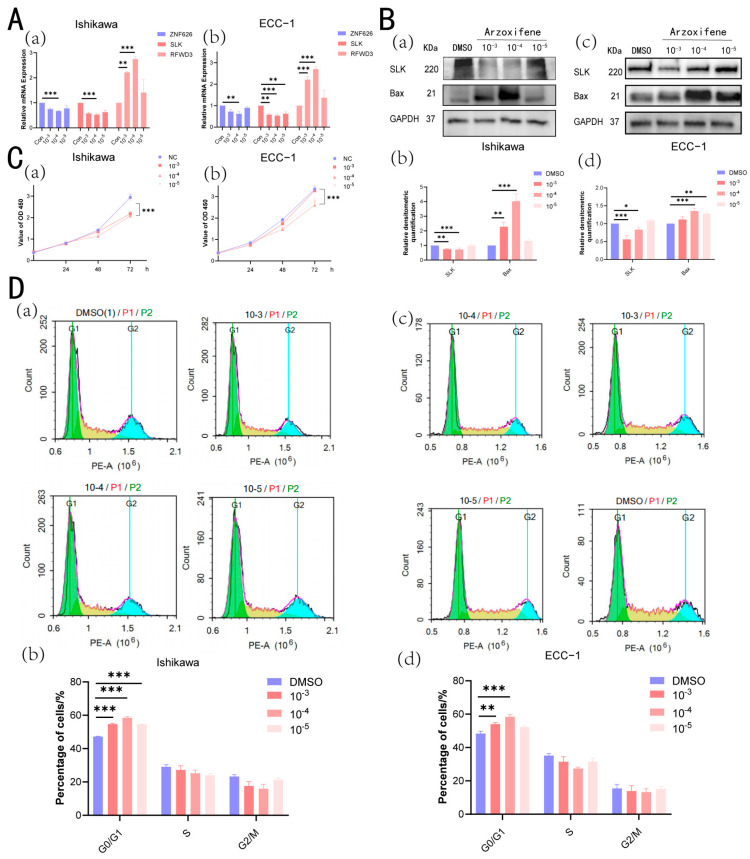
Arzoxifene restrains the proliferation and apoptosis of Ishikawa cells and ECC-1 cells. (**A**) The relative expression levels of ZNF626, SLK, and RFWD3 in Ishikawa cells (**a**) and ECC-1 cells (**b**) were assessed using qRT-PCR following stimulation with various concentrations of Arzoxifene (0 [control], 10^−3^ mol/L, 10^−4^ mol/L, and 10^−5^ mol/L) for 72 h. (**B**) Western blot analysis of SLK and Bax expression levels in Ishikawa cells (**a**) and ECC-1 cells (**c**) following 72 h of stimulation with different Arzoxifene concentrations. Corresponding histograms present the quantified Western blot results for Ishikawa cells (**b**) and ECC-1 cells (**d**). (**C**) CCK8 assays demonstrated the effects of different Arzoxifene concentrations on the proliferation of Ishikawa cells (**a**) and ECC-1 cells (**b**) at 24, 48, and 72 h. (**D**) The percentage distribution of cells across different cell cycle phases in Ishikawa cells (**a**) and ECC-1 cells (**c**) after 72 h of stimulation with varying Arzoxifene concentrations. A histogram illustrating the average percentages of cells in the G0/G1, S, and G2/M phases for both control and treated groups in Ishikawa cells (**b**) and ECC-1 cells (**d**). Values represent the mean ± S.E.M. of a minimum of three independent experiments. Significance is calculated by performing an unpaired *t*-test between the control and treated groups: * *p* < 0.05; ** *p* < 0.01; *** *p* < 0.001.

Given the role of E2 in promoting cell proliferation, we further investigated whether Arzoxifene, a selective estrogen receptor modulator (SERM), could counteract this effect. After stimulating the Ishikawa cell line with different concentrations of Arzoxifene for 72 h, a dose-dependent decrease in ZNF626 and SLK mRNA expression was observed, most pronounced at 10^−4^ mol/L. Conversely, RFWD3 exhibited increased mRNA expression, also most pronounced at 10^−4^ mol/L (Figure 7A). At the protein level, we similarly validated the decrease in SLK expression, showing no significant change at 10^−5^ mol/L and the most pronounced decrease at 10^−4^ mol/L (Figure 7B). Cell proliferation assays showed that 10^−3^ mol/L and 10^−4^ mol/L of Arzoxifene significantly inhibited hyperplasia, while 10^−5^ mol/L had no effect (Figure 7C). Similarly to E2, the middle concentration (10^−4^ mol/L) exhibited the strongest response (Figure 7A–D), possibly due to a biphasic effect, where moderate doses elicit the optimal response, while higher doses may induce feedback inhibition or cytotoxicity. Further analysis revealed that Arzoxifene treatment led to a notable increase in the G0/G1 phase, confirming its inhibitory effect on cell proliferation. At 10^−4^ mol/L, Bax expression increased approximately fourfold, indicating enhanced apoptosis (Figure 7D). ECC-1 cells exhibited similar effects; however, the tumorigenic mechanisms of the two cell lines differ, leading to distinct responses. The concentrations of Arzoxifene required to induce significant changes in gene expression and alterations in cell cycle and apoptosis also varied between Ishikawa and ECC-1 cells, as observed with E2 stimulation. Overall, under appropriate concentrations of Arzoxifene, it modulates gene expression to influence cell proliferation, cycle progression, and apoptosis.

## 4. Discussion

In this two-sample Mendelian randomization (MR) study, we comprehensively elucidated genes associated with endometrial cancer, identified genes with clear causal relationships to the disease, and explored the potential biological connections between these genes and immunoinflammation. To further clarify the role of the association between relevant genes and estrogen in endometrial cancer, we conducted in vitro validations by sequentially applying estrogen and estrogen receptor antagonists to EC cells. Results indicated that estrogen promotes the hyperplasia of EC cells by either upregulating or downregulating relevant genes.

By cross-referencing the 1368 differentially expressed genes obtained from a public database within the training set with the 157 genes identified through MR analysis, we found that seven genes—ZNF544, ZNF626, SLK, HIGD2A, RFWD3, C5, and SECTM1—were significantly associated with endometrial cancer. The two-sample MR analysis identified ZNF626 and SLK as facilitating factors of EC progression, while RFWD3 was found to be a protective factor. Although the roles of ZNF626, SLK, and RFWD3 in endometrial cancer have not been extensively discussed, their biological functions in relation to other tumors are well documented and can provide insights for our study. ZNF626 encodes a zinc finger protein that plays a role in regulating gene transcription and may impact cell proliferation through this regulatory pathway [22]. The mechanism in the upregulation of ZNF626 in endometrial cancer may involve its role as a regulator, influencing the growth and apoptosis of cells. While the specific mechanisms remain under investigation, ZNF626 promoting cancer occurrence and development warrants further exploration. Ste20-like kinase (SLK) represents a critical subgroup of kinases involved in cell hyperplasia, relocation, and polarization [23,24,25,26]. SLK has been reported to induce apoptosis by activating caspase-3 [27]. However, its role in apoptosis appears to be context dependent. In certain cancers, SLK overexpression has been associated with increased cell proliferation, protein reorganization in muscle tissue, and enhanced tumor invasion and metastasis [28,29]. These findings suggest that SLK may exhibit dual roles, promoting apoptosis under specific conditions while facilitating tumorigenicity in others. This context-specific behavior warrants further investigation to fully understand its role in cancer progression. The repair of damaged DNA is vital in preventing the development of various cancers. RFWD3, a potential tumor suppressor, functions as an ubiquitin E3 ligase and plays a key role in DNA replication, DNA damage repair, and the effective termination of these processes [30,31,32,33,34]. Interestingly, the reduced expression of RFWD3 has been associated with a better prognosis in certain cancer contexts [35,36]. This paradox may reflect the dual roles of RFWD3 reported in the literature, where its function could be context dependent. For example, while RFWD3’s role in DNA repair generally supports genomic stability, in certain cancers, its activity might contribute to the survival of damaged cells, facilitating tumor progression. Such context-specific roles warrant further investigation to fully understand RFWD3’s contribution to cancer biology. Studies have shown that during DNA damage, RFWD3 is recruited and may play a more direct role in DNA damage repair through its interaction with replication protein A (RPA) [37]. RFWD3 is also closely associated with the DNA damage response (DDR) and Fanconi anemia (FA) signaling pathways, which coordinate various DNA repair mechanisms [38]. Therefore, ZNF626, SLK, and RFWD3 may represent potential therapeutic targets for endometrial cancer. The findings offer some new ideas and gene loci for understanding the role of specific tumor-associated genes in EC and their hidden impacts on clinical treatment.

Our results indicate that estrogen can upregulate the ZNF626 and SLK genes while downregulating the RFWD3 gene. There is a close association between estrogen and changes in genes that promote cell proliferation and inhibit apoptosis, which may represent a prospective mechanism of EC. Excess estrogen and insulin, along with progesterone deficiency, are considered primary hormonal imbalances associated with EC, increasing the risk of epithelial cell transformation/carcinogenesis [39,40,41,42,43]. Numerous studies have confirmed that prolonged exposure to estrogen is significantly associated with an increased risk of hormone-related cancers, including endometrial cancer [9,44,45,46]. Our study innovatively proposes that the upregulation of ZNF626 and SLK genes and the downregulation of the RFWD3 gene—both mediated by estrogen—promotes the progression of EC. Considering the intricate complexity of cellular signaling networks, the expression of downstream genes can be mediated by various factors beyond estrogen, indicating that antagonizing estrogen alone may not prevent the onset of endometrial cancer. In populations where estrogen-targeted antagonistic therapies are ineffective, targeting the regulation of estrogen downstream genes ZNF626, SLK, and RFWD3 may yield beneficial therapeutic outcomes. Our results suggest that these effects are most pronounced at an estrogen concentration of 10^−8^ mol/L, providing a reference for the further stratification of endometrial cancer based on estrogen.

Further relevance analysis between genes and immunization revealed a decreased proportion of memory CD4+ T cells in the tumor group, with RFWD3 positively correlating with M1 macrophages, ZNF626 positively correlating with M2 macrophages, and SLK negatively correlating with M1 macrophages. This indicates that tumor cells may reduce the body’s immune capacity through mechanisms including immunological escape, alterations in the immune microenvironment, and exhaustion due to chronic antigen stimulation. The tumor microenvironment is important in promoting tumor cell proliferation under unopposed estrogen stimulation. The density of macrophages and lymphocytes in EC is higher than that in benign endometrial tissue, promoting tumor growth and invasion through the release of various cytokines and chemokines [47,48]. Consistent with our findings, studies indicate that M1 macrophages, characterized by cytotoxic potential, are considered to have an antitumor phenotype, whereas M2 macrophages, associated with repair, are denoted as having a tumor-promoting phenotype [48]. Balancing immune cells within the tumor microenvironment may represent a potential treatment direction in the near future.

Nevertheless, this study has certain limitations. For instance, the mechanisms through which estrogen regulates the expression of ZNF626, SLK, and RFWD3 genes remain unclear. Additionally, the pathways by which these core genes influence cell proliferation and apoptosis lack in vivo experimental validation. Therefore, the detailed molecular mechanisms by which E2 affects EC require further elucidation.

EC results from the interplay of estrogen, genetic factors, and the tumor inflammatory microenvironment. Estrogen promotes cell proliferation and inhibits apoptosis by upregulating ZNF626 and SLK genes and downregulating the RFWD3 gene, while also leading to a decline in memory CD4+ T cells and a shift in macrophage phenotypes from M1 to M2 within the tumor inflammatory microenvironment. This interplay further contributes to the development and progression of EC.

## 5. Conclusions

We identified significant differentially expressed genes (DEGs) implicated in the pathogenesis of EC through a combination of GEO database analyses and Mendelian randomization (MR). Functional investigations elucidated the associated signaling pathways, highlighting their enriched biological processes and molecular activities. In vitro experiments further demonstrated that estrogen enhances cellular proliferation and suppresses apoptosis by upregulating ZNF626 and SLK while concurrently downregulating RFWD3. Additionally, immune infiltration analysis revealed that these DEGs might remodel the tumor microenvironment by modulating memory CD4+ T cells and inducing a macrophage phenotype transition from M1 to M2. These alterations collectively contribute to the initiation and progression of EC.

## Figures and Tables

**Table 1:** Primer sequences used for quantitative real-time PCR analysis.

Gene	Forward Sequence (5′-3′)	Reverse Sequence (5′-3′)
GAPDH	GGGTCGGTGTGAACGGATTTGG	GCCGTGGGTAGAGTCATACTGGAAC
ZNF626	GCCAAACCCTCAGTAATGTGT	TGTGCACCTTACACTCATCCA
SLK	ATCGCTTGCGAGATGAAGCC	TTGTGCAAGCTCCTCTTTCCT
RFWD3	AGTTGGCGTAGGTGCATTCG	AGCCATCACTGAAACCAGCA

## Data Availability

The original data used in this study were from public databases, including GEO database, GWAS database, and eQTL Gen alliance. All data are strictly in accordance with the terms of use of the relevant database, only for academic research, and do not involve any identifiable personal information, so no additional ethical approval is required. In addition, other data and analysis results generated in this paper are included in the article and Supplementary Materials. For further information, please contact the corresponding author.

## Supplementary Materials

The following supporting information can be downloaded at: https://www.mdpi.com/article/10.3390/biomedicines13020498/s1, Figure S1: Supplementary Figure S1, the blots of all bands and molecular weight markers; Tables S1–S10: Supplementary Tables S1–S10.
