# Supplementary material for: Estrogen Promotes Endometrial Cancer Development by Modulating ZNF626, SLK, and RFWD3 Gene Expression and Inducing Immune Inflammatory Changes
Source: Biomedicines. 2025 Feb 17;13(2):498. doi: 10.3390/biomedicines13020498 (PMC11853163; doi:10.3390/biomedicines13020498)
Supplement: Supplementary file 1 [file biomedicines-13-00498-s001.zip › Supplementary Figure S1, the blots of all bands and molecular weight markers.pdf]

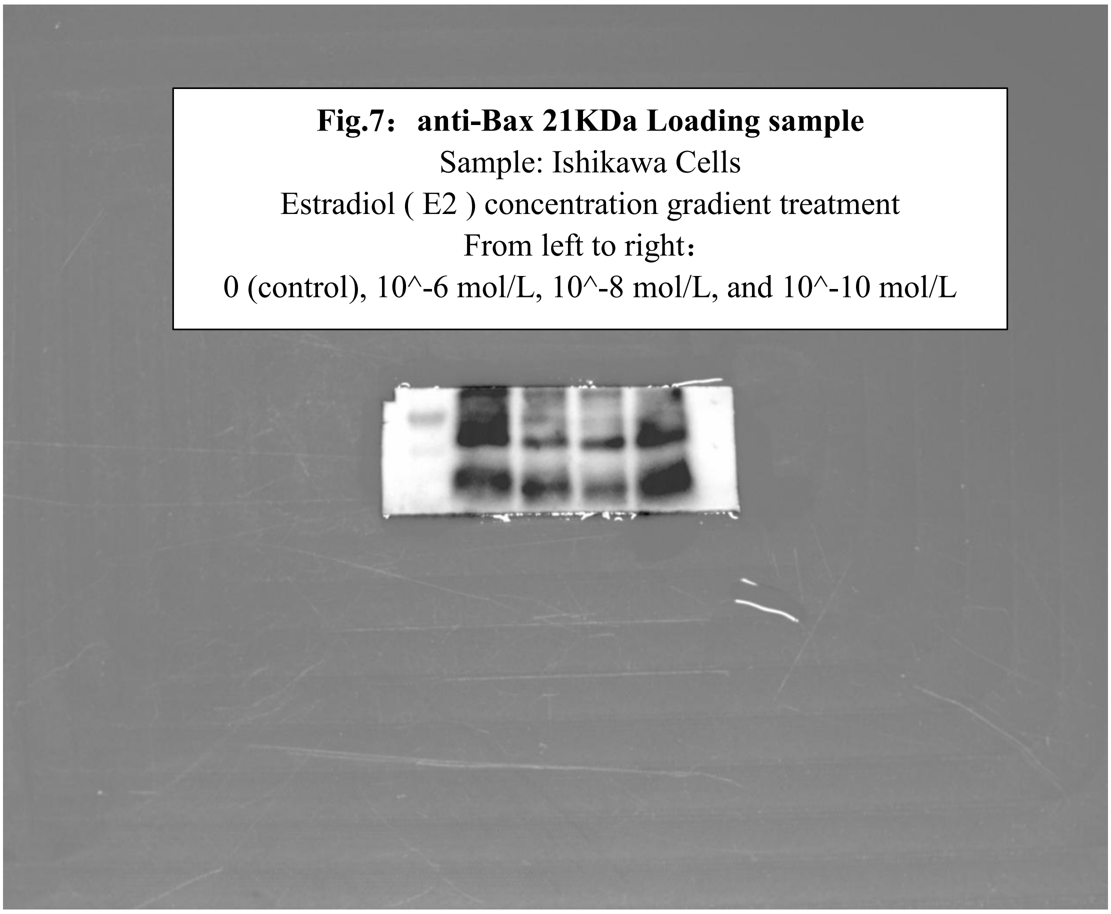

Fig.7：anti-Bax 21KDa Loading sample
Sample: Ishikawa Cells
Estradiol (E2) concentration gradient treatment
From left to right：
0 (control), 10^-6 mol/L, 10^-8 mol/L, and 10^-10 mol/L

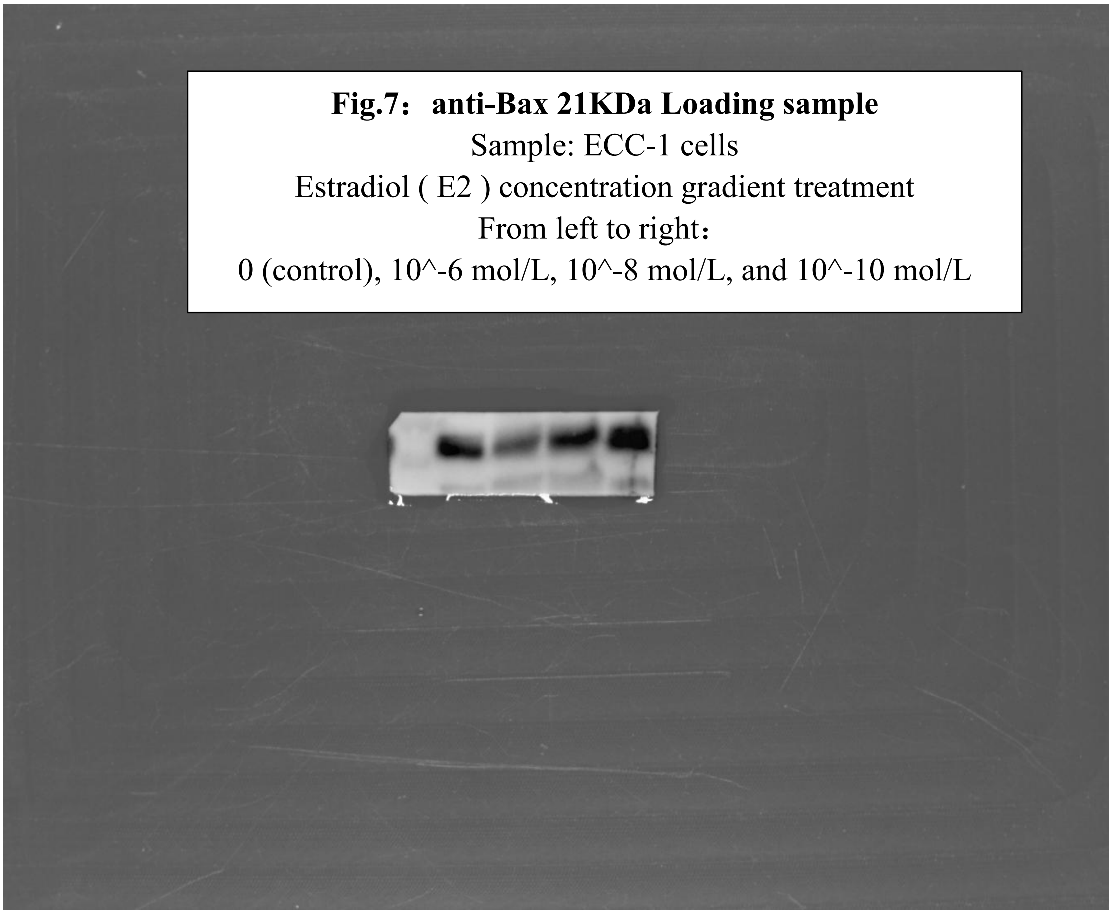

Fig.7：anti-Bax 21KDa Loading sample
Sample: ECC-1 cells
Estradiol (E2) concentration gradient treatment
From left to right：
0 (control), 10^-6 mol/L, 10^-8 mol/L, and 10^-10 mol/L

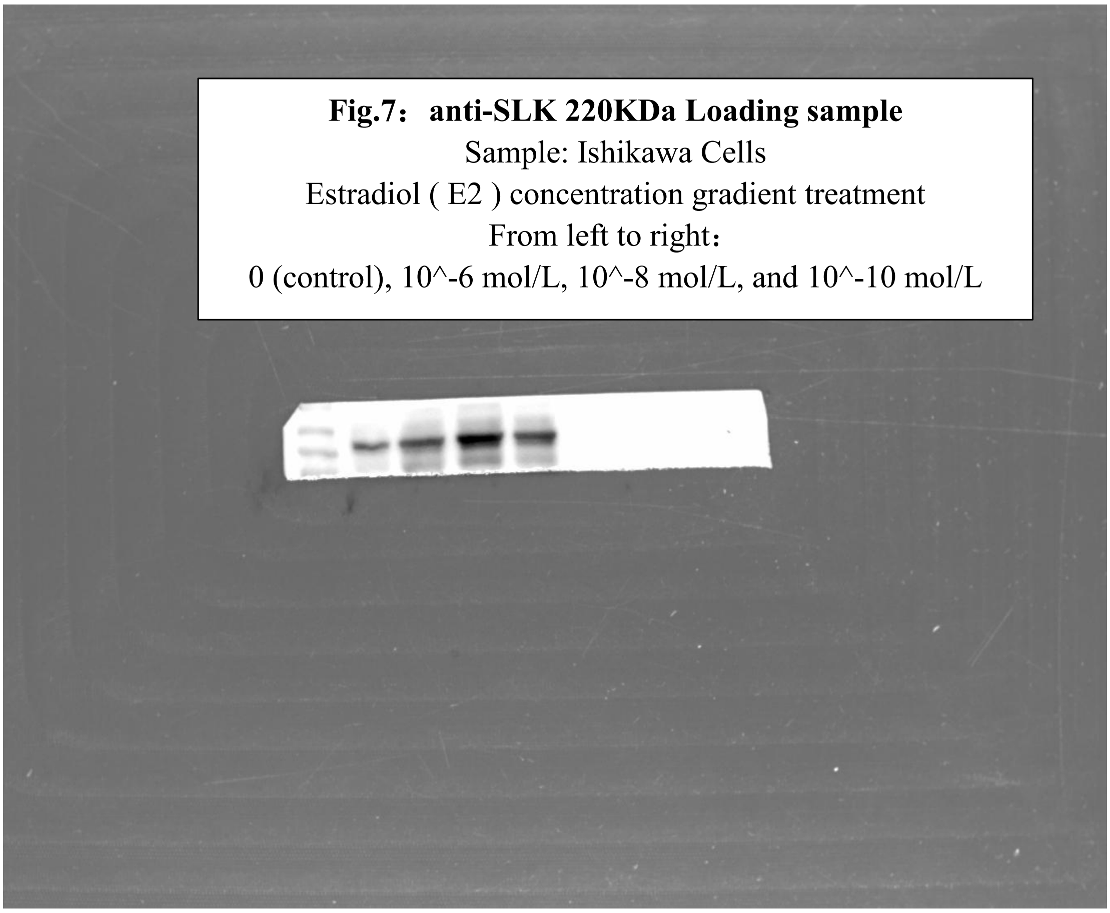
Fig.7：anti-SLK 220KDa Loading sample
Sample: Ishikawa Cells
Estradiol (E2) concentration gradient treatment
From left to right：
0 (control), 10^-6 mol/L, 10^-8 mol/L, and 10^-10 mol/L

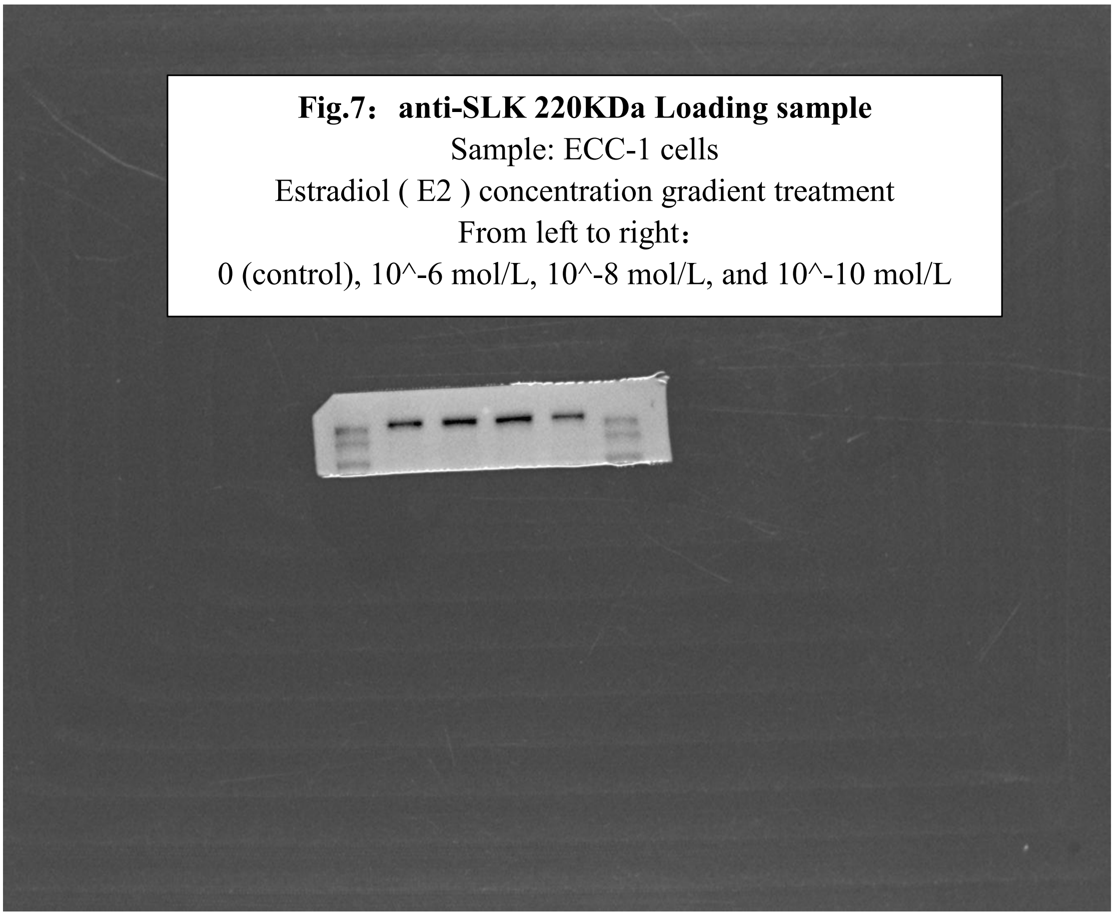
Fig.7：anti-SLK 220KDa Loading sample
Sample: ECC-1 cells
Estradiol (E2) concentration gradient treatment
From left to right：
0 (control), 10^-6 mol/L, 10^-8 mol/L, and 10^-10 mol/L

Fig.7：anti-GAPDH 37KDa Loading sample
Sample: Ishikawa Cells
Estradiol (E2) concentration gradient treatment
From left to right：
0 (control), 10^-6 mol/L, 10^-8 mol/L, and 10^-10 mol/L

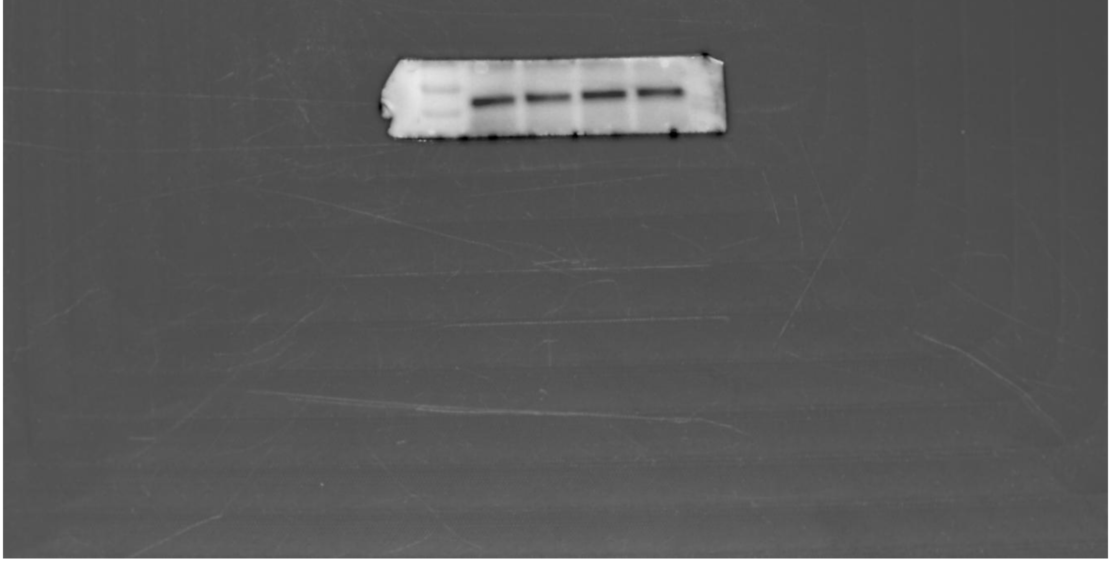

Fig.7：anti-GAPDH 37KDa Loading sample
Sample: ECC-1 cells
Estradiol (E2) concentration gradient treatment
From left to right：
0 (control), 10^-6 mol/L, 10^-8 mol/L, and 10^-10 mol/L

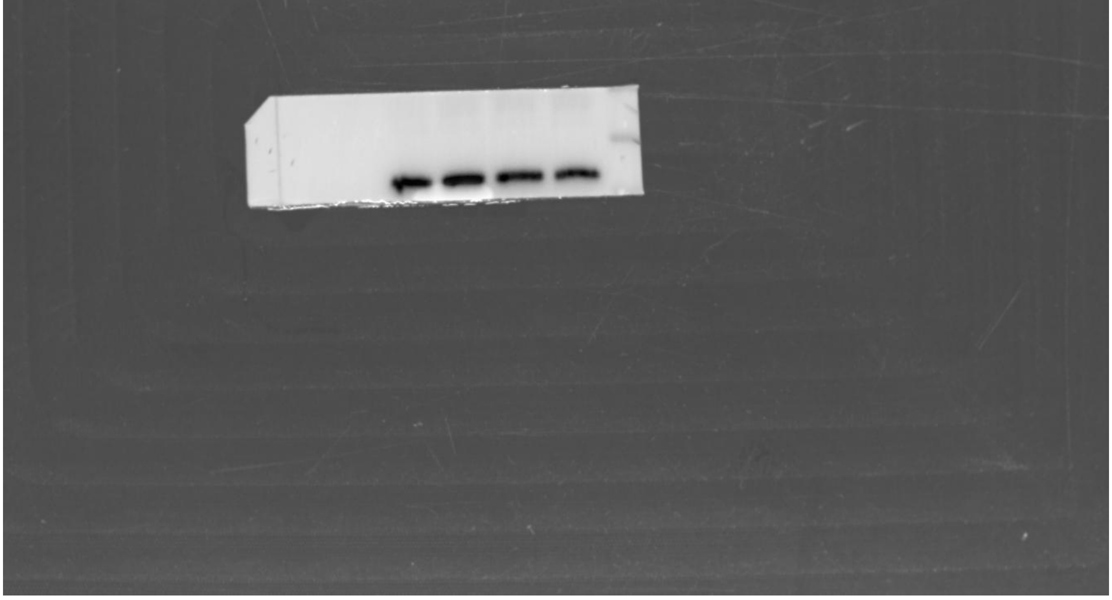

Fig.8：anti-Bax 21KDa Loading sample

Sample: Ishikawa Cells

Arzoxifene concentration gradient treatment

From left to right：

0 (control), 10^-3 mol/L, 10^-4 mol/L, and 10^-5 mol/L

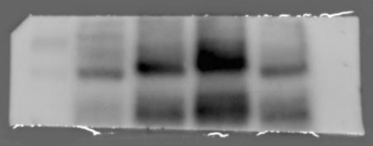

Fig.8：anti-Bax 21KDa Loading sample

Sample: ECC-1 cells

Arzoxifene concentration gradient treatment

From left to right：

0 (control), 10^-3 mol/L, 10^-4 mol/L, and 10^-5 mol/L

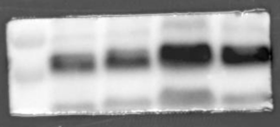

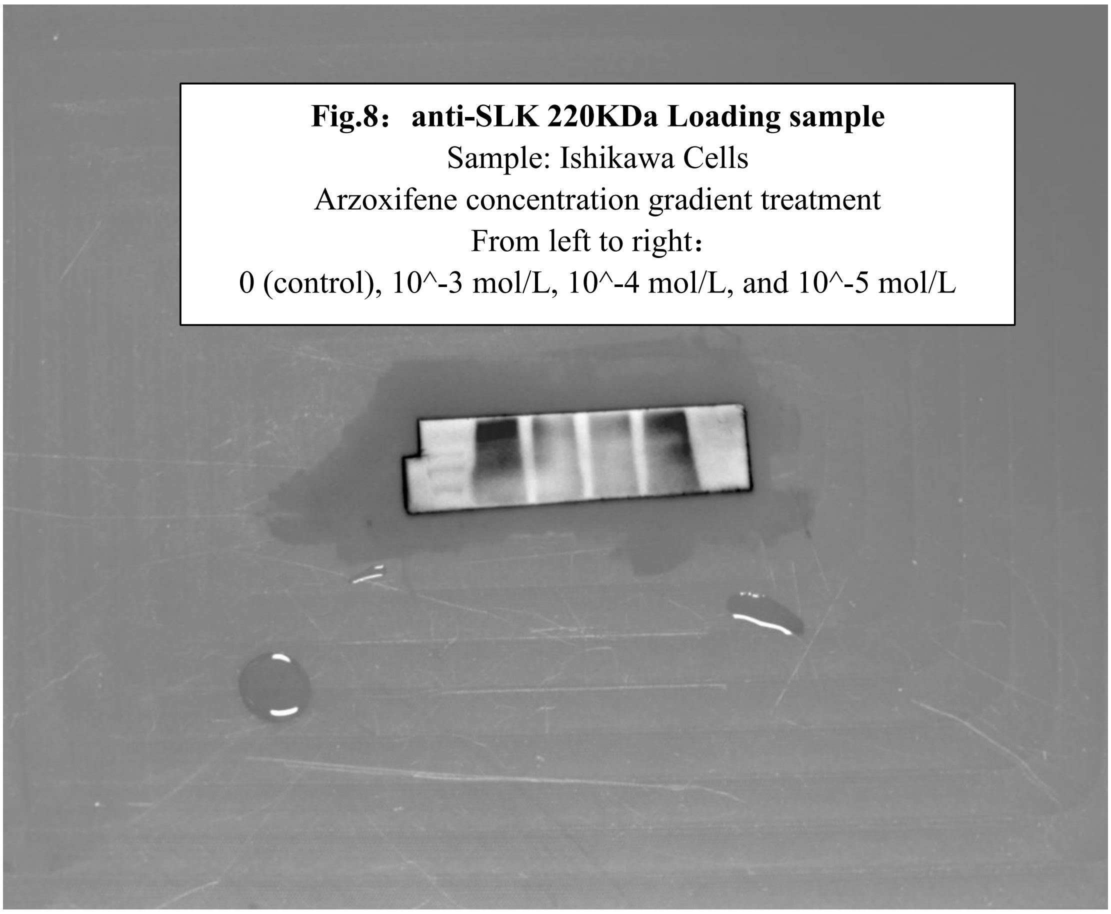

Fig.8：anti-SLK 220KDa Loading sample
Sample: Ishikawa Cells
Arzoxifene concentration gradient treatment
From left to right：
0 (control), 10^-3 mol/L, 10^-4 mol/L, and 10^-5 mol/L

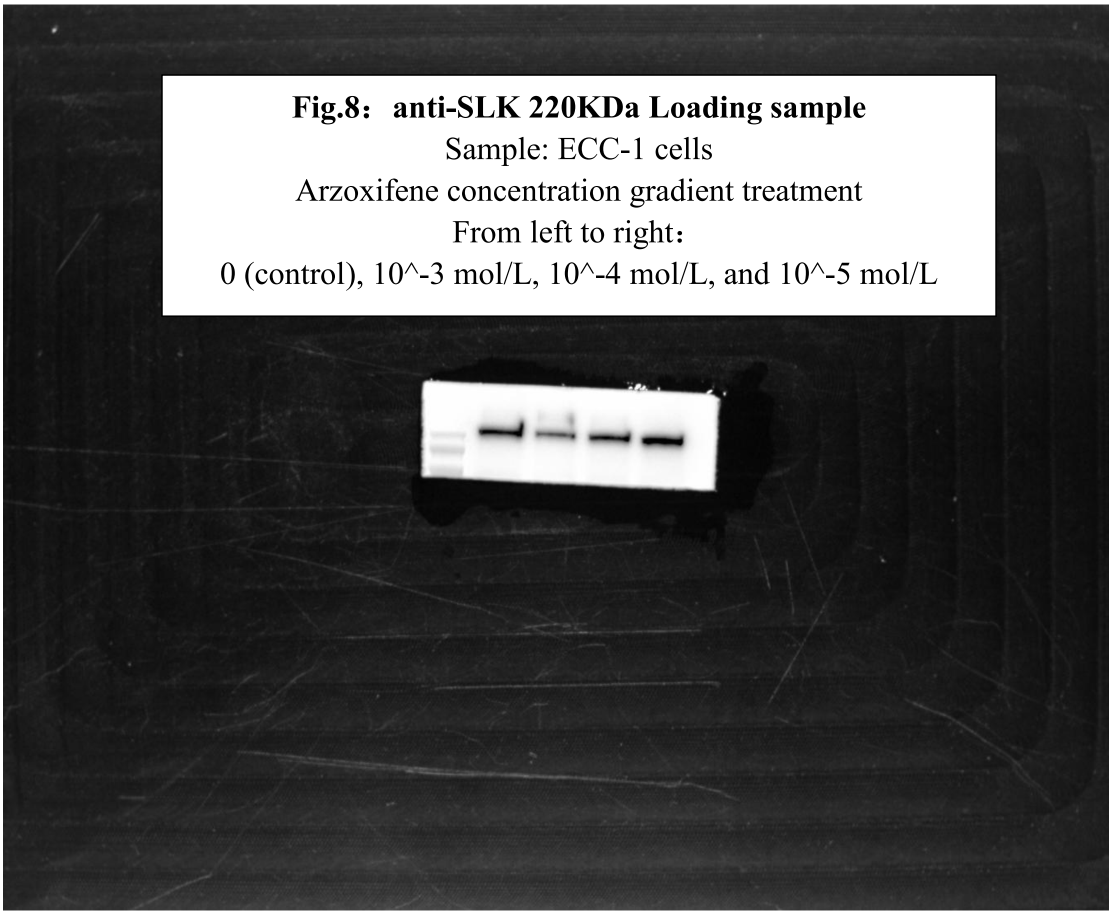

Fig.8：anti-SLK 220KDa Loading sample
Sample: ECC-1 cells
Arzoxifene concentration gradient treatment
From left to right：
0 (control), 10^-3 mol/L, 10^-4 mol/L, and 10^-5 mol/L

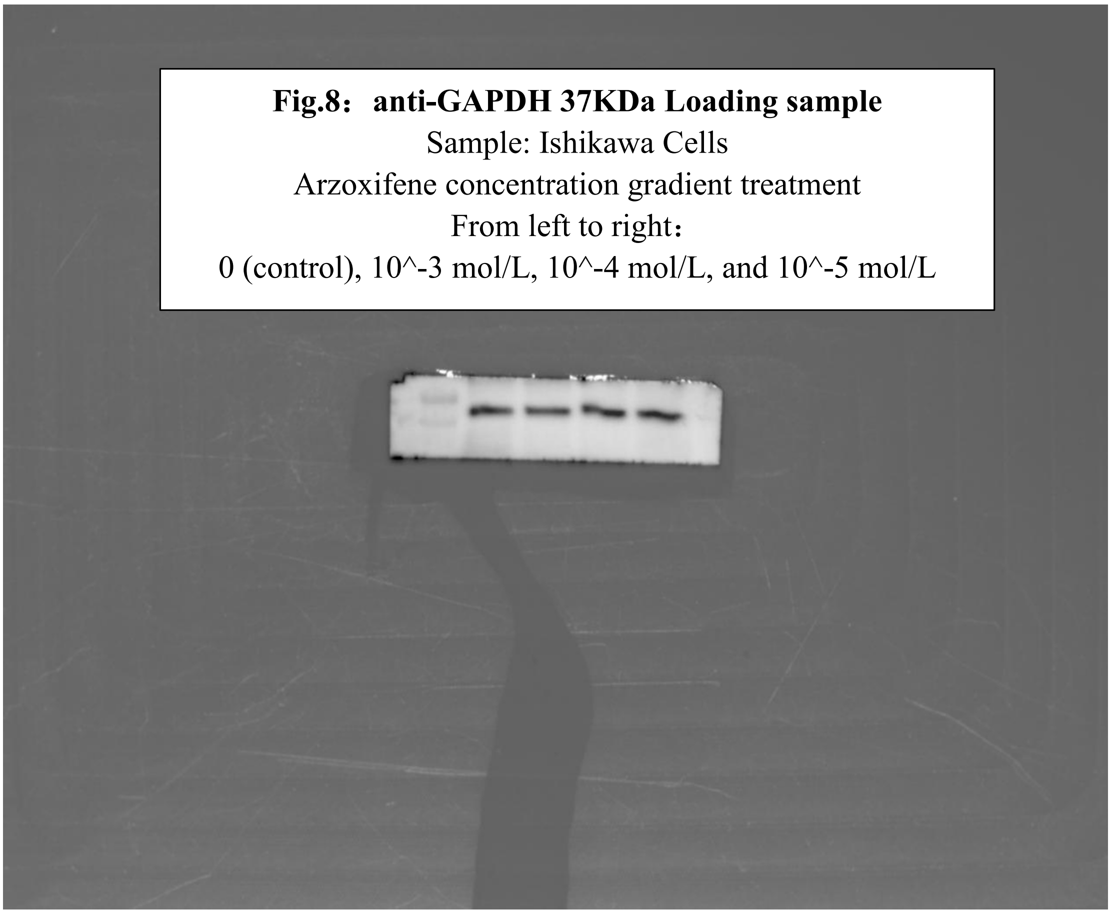

Fig.8：anti-GAPDH 37KDa Loading sample
Sample: Ishikawa Cells
Arzoxifene concentration gradient treatment
From left to right：
0 (control), 10^-3 mol/L, 10^-4 mol/L, and 10^-5 mol/L

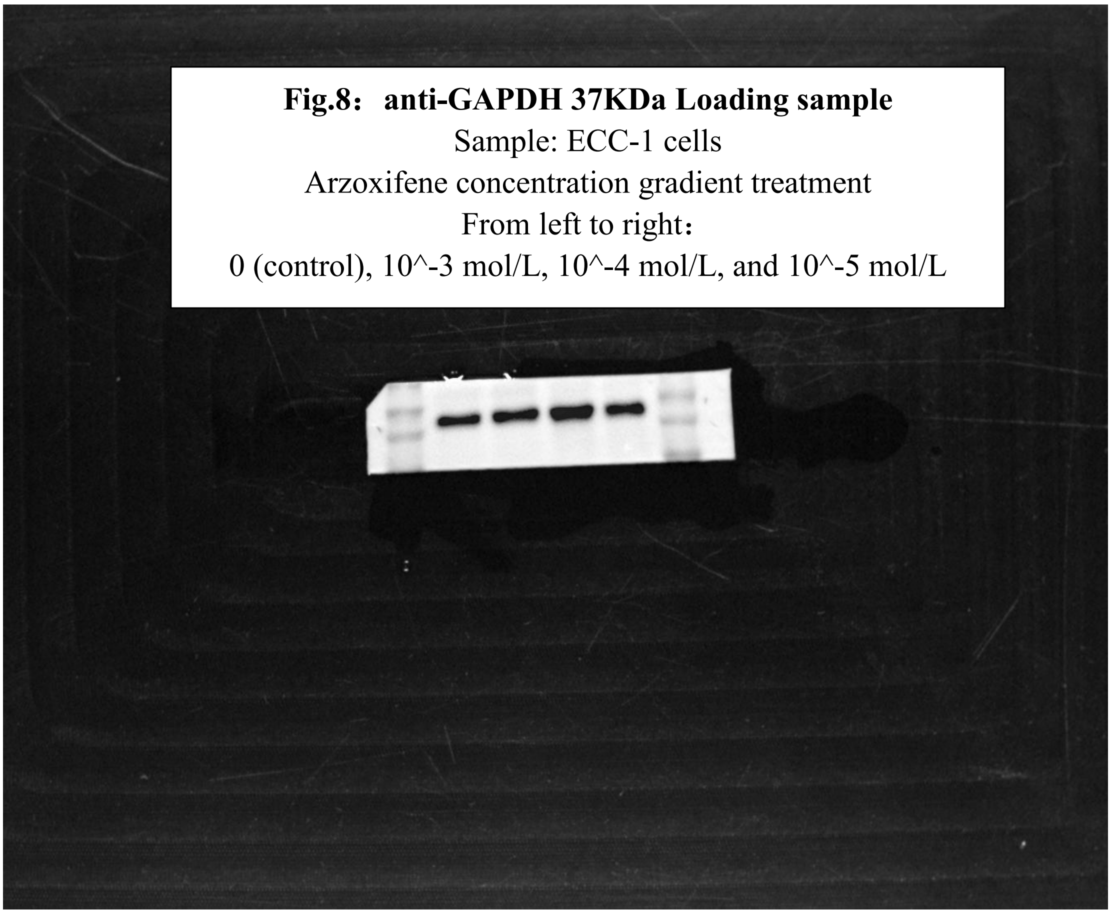

Fig.8：anti-GAPDH 37KDa Loading sample
Sample: ECC-1 cells
Arzoxifene concentration gradient treatment
From left to right：
0 (control), 10^-3 mol/L, 10^-4 mol/L, and 10^-5 mol/L
